# A Survey of Rural Residents’ Perception and Response to Health Risks from Hot Weather in Ethnic Minority Areas in Southwest China

**DOI:** 10.3390/ijerph16122190

**Published:** 2019-06-21

**Authors:** Haipeng Li, Jiabao Guan, Hui Ye, Haichen Yang

**Affiliations:** School of Public Management, South-Central University for Nationalities, Wuhan 430074, China; lihaipeng@mail.scuec.edu.cn (H.L.); 2017110085@mail.scuec.edu.cn (J.G.); 2017110091@mail.scuec.edu.cn (H.Y.)

**Keywords:** hot weather, health risk, perception, response, ethnic minority areas, southwestern China

## Abstract

Ethnic minority areas in southwestern China are facing frequent high-temperature heatwaves. The health risk perceptions and responses of the local residents need to be investigated in order to formulate public policies to mitigate the impacts of climate change on health. In this study, a household survey was conducted in Pengshui Miao and Tujia Autonomous County of Chongqing from January to February 2019. A total of 624 local residents were sampled using the multi-stage sampling method. We used multivariate logistic regression models to explore the factors affecting risk perceptions and responses with regard to hot weather. The results showed that despite a relatively high level of risk perception, the study population had a very low level of willingness to see a doctor (24.4%), especially ethnic minority residents (17.5%). In particular, 80% of residents were aware of climate warming and 79% of residents were aware of the health risks of hot weather. Almost all survey participants reported a response to hot weather, with more than half of the participants stating that they would go somewhere cooler (58.5%) and drink more water (56.3%). Compared with the Han Chinese, ethnic minority participants had a higher perception of warm temperature (*p* <0.001) and associated health risks (*p* <0.001) but a lower perception of physical discomfort (*p* <0.001) and aggravated diseases (*p* = 0.001). The logistic models indicated that ethnic minority, residence time, outdoor working hours, and health status can significantly influence perceptions and subsequently significantly affect coping behaviors. In conclusion, our findings provide significant implications for the development of policies and health education and promotion programs for ethnic minorities in southwest China to aid them in maintaining good health during future hot weather events.

## 1. Introduction

In the 2014 World Health Organization’s Health and Climate Conference and the United Nations Climate Summit it was reported that climate change posed a major threat to human health, but very few countries have considered health issues when formulating climate change policies. As a result of climate change, hot weather will become more frequent and intense [1]. According to data from the World Meteorological Organization (WMO), the average global temperature in the first 10 months of 2018 was the hottest year since meteorological records began, at about 1.8 degrees Celsius higher than that in the late 19th century. With the frequent occurrence of hot weather, the prevalence, hospital visit rate, and mortality rate of weather-related medical conditions have increased significantly. This shows that hot weather is an important risk factor that threatens human health [2,3].

Many studies have shown that to effectively cope with disasters from hot weather, it is necessary to improve residents’ perceptions of the health hazards caused by hot weather and adopt adaptive measures under cognitive guidance [4,5]. Since the 1990s, developed countries such as the United States and United Kingdom have conducted public surveys on global warming and heatwaves [6,7], which have played a positive role in the formulation of relevant policies. In recent years, perception research has mainly focused on the investigation of vulnerable groups (such as the elderly, women, children, hospital patients, etc.) [8,9]. However, despite the indication that race and immigration status are associated with differences in heat perception, research on different ethnicities, immigrants, and ethnic groups is obviously lacking. A study on ethnic differences in calorie-related deaths in four U.S. cities found that mortality among black individuals is more closely related to hot weather [10]. Similarly, a study of vulnerable populations in California also found differences in the risk of heat-related diseases among different ethnic groups [11]. In addition, Australian studies have shown that Aboriginal people living in remote areas show vulnerability to hot weather [12]. Multiple studies have stated that the number of heat-related deaths among immigrants has increased in recent years [13,14]. In addition, due to differences in language, living environment, economic conditions, culture, psychology, and even genetic differences, the ability to combat hot weather and heat-related diseases among different races and ethnic groups is also quite different [15,16,17,18].

Southwest China contains the five southwestern provinces (autonomous regions and municipalities) with a total area of 2.5 million square kilometers, accounting for 24.5% of China’s land area [19]. Geographically, it includes the southeastern part of the Qinghai–Tibet Plateau, the Sichuan Basin, and most of the Yunnan–Guizhou Plateau. The geographical location of the area is between 97°21’~110°11’E longitude and 21°08’~33°41’N latitude. Corresponding to the terrain, its climate is divided into three categories: the humid north subtropical monsoon climate in the Sichuan Basin, the subtropical monsoon climate in the Yunnan–Guizhou Plateau, and the unique and diverse plateau climate of the Qinghai–Tibet Plateau. The southwestern region has the largest number of ethnic minorities in China including the Bai, Dai, Shui, Wa, Miao, Nu, Menba, Yi, and Tujia. The Pengshui County of Chongqing is located on the southeastern edge of the Sichuan Basin. Its terrain is dominated by low mountains and hills at an altitude of 1000–1800 m with a flat dam. When the temperature rises, the mountain rock and soil easily emit heat after passing through the sunshine, and there is a mountain block around it, which is not conducive to the diffusion of heat and easily causes the temperature to rise [14,20]. There is growing evidence that hot weather is affecting the lives of local minority residents. In light of this, to study the ways in which minority residents cope with hot weather and the health risks, this paper explored the factors affecting perception and response to health risks from hot weather in Pengshui Miao and Tujia Autonomous County of Chongqing.

## 2. Materials and Methods

Cross sectional surveys were conducted in Pengshui Miao and Tujia Autonomous County of Chongqing (Pengshui County for short) from January to February 2019. We used a multi-stage sampling method to randomly select local residents. After the descriptive analysis, multivariate logistic regression models were developed to explore the factors affecting risk perceptions and responses with regard to hot weather. All analyses were performed by STATA v14 with a significance level of 0.05.

### 2.1. Study Population

Pengshui Miao and Tujia Autonomous County (107°48′E–108°36′E, 28°57′N–29°51′N) are located southeast of Chongqing City, on the southeastern edge of the Sichuan Basin. Pengshui County has an administrative area of 3903 square kilometers and administers three streets, 36 townships, and 296 villages with a registered population of 703,000. There are 11 ethnic minorities such as the Miao and Tujia in the county, accounting for 63.9% of the total population. Pengshui County belongs to the mid-subtropical warm monsoon climate zone. The average annual temperature is 17.5 °C, the average annual rainfall is 1104.2 mm, the annual average evaporation is 950.4 mm, and the annual average pressure is 978.6 hPa. The summers are hot and dry, and days with temperatures above 35 °C are mainly concentrated in July and August. 

**Figure 1:**
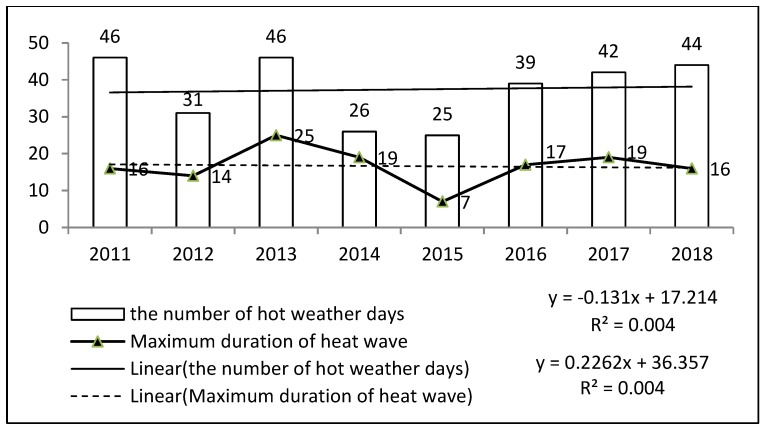
Number of hot weather days and maximum heatwave duration in the administrative center of Pengshui County in July and August from 2011 to 2018. Note: ^1^ Hot weather was defined as a maximum daily temperature of more than 35 °C, and heatwave was defined as hot weather continuing for more than three days; ^2^ The data source was the daily maximum temperature data of the National Meteorological Information Center of China (http://data.cma.cn/).

The statistical data on hot weather days and heatwave duration in Pengshui County from 2011 to 2018 (Figure 1) show that the number of hot weather days was 299, accounting for 10.2% of this eight-year period, with an average of 37 high-temperature days/year. The distribution of high-temperature days in Pengshui County has been very different over the past few years. The highest values occurred in 2011 and 2013, when there were 46 high-temperature days. The lowest value occurred in 2015, although there were still 25 high-temperature days. In the past eight years, there have been heatwaves with a hot weather lasting for more than three days, and the longest lasted for over 25 days. Thus, it is clear that hot weather, especially long-term heatwave events, often occurs in Pengshui County. The trend line shows that the number of hot weather days and the duration of heatwaves have increased year by year, consistent with the occurrence of global warming, in particular, the number of hot weather days in 2018 was 44, which was the year with the most hot days except for 2011 and 2013.

### 2.2. Survey and Sampling

The research data in this paper came from a questionnaire survey conducted by the research team from January to February 2019 in Pengshui Miao and Tujia Autonomous County, Chongqing. The survey was completed by rural residents of the county who were selected using the multi-stage sampling method: First, six of the 36 townships in the county were selected according to the simple random sampling method. Then, a wealthy village and a poor village were selected from each of the six sample townships. Finally, using the cluster sampling method, local residents were selected from the 12 villages; 720 questionnaires were distributed, and 624 valid questionnaires were collected. Thus, the effective recovery rate was 86.7%, with ethnic minority residents accounting for 61.2% of the sample. The questionnaire surveyed the family population and economic situation, the respondents’ perceptions of hot weather, and the responses of respondents to hot weather.

### 2.3. Statistical Analyses

Perceptions of health risk from hot weather were measured from four aspects: perception of warmer temperature (Do you think the weather has been getting warmer in recent years in your village?), perception of associated health risks (Do you think that high temperatures are a threat to human health?), perception of physical discomfort during hot weather (Do you feel that higher temperatures have caused physical discomfort of yourself?), and perception of aggravated diseases during hot weather (Do you feel that higher temperatures have increased the primary disease and the deterioration?). For each, if the respondent was aware of the aspect, the variable assignment was 1. Otherwise, it was 0.

Responses to hot weather were measured from three aspects: seeing a doctor (Will you see a doctor if you have perceived physical discomfort during hot weather?), taking adaptive actions (Do you take any of the following actions to respond to hot weather, such as drinking extra water, going to somewhere cooler, staying indoors?), and asking for help (Whether you will ask for help if you are affected by extreme heat events?). For each of the above aspects, if coping behavior was present, the variable assignment was 1. Otherwise, it was 0.

A descriptive analysis of the proportions of the perception and responses was conducted, and the situation across the whole study population was estimated with 95% confident intervals (CIs). Differences in responses among ethnic minorities and the Han minority were compared by chi-squares tests. Four separate multivariate logistic models were developed to examine the potential factors that could affect the perceptions of warm temperature, health risks, physical discomfort, and disease aggravation, and three separate models were developed to assess whether the perceptions could affect the adoption of different coping behaviors. The relationship between variables was estimated by Odds Ratio (OR), and Robust Standard Error (Robust Std. Err.) was used to measure the significance of logistic models.

### 2.4. Ethics Approval

Informed consent was taken from the participants and the study was approved by the Ethics Committee of South-Central University for Nationalities in China in 2017 (Grant number 2017-SCUEC-MEC-008).

## 3. Results

### 3.1. Descriptive Analyses

The majority (61.2%) of the participants were ethnic minorities including the Miao and Tujia. The average age was 43 and 33.7% of respondents were females. The average residence time of the participants was 39 years. A total of 58.2% had received an education beyond high school, and 73.6% were married. A total of 34.3% of participants were employed, and 36.2% worked or stayed outdoors for no less than seven hours a day in July and August. Twelve percent of participants reported a generally poor health status (for Han 11% and for the ethnic minority 12.6%), and 10.3% had an impairment or disability with restricted movement (for Han 12.8% and for the ethnic minority 8.6%). A total of 25% had a chronic disease (for Han 9.1% and for the ethnic minority 35.1%) (Table 1).

The majority of the participants (80%) were aware of warm temperature and associated health risks (79%). A total of 92% of participants reported physical discomfort during hot weather and 35% perceived that diseases are aggravated during hot weather. However, 24.4% would see a doctor if they perceived physical discomfort during hot weather, most respondents (98.7%) stated that they could take actions to respond to hot weather, and 91% would ask for help if affected by extreme weather events (Table 2).

We further analyzed the differences in perceptions and responses among the different ethnic groups. Table 2 shows that ethnic minority participants (61.2% of the study population) were more aware of warm temperature and their associated health risks, but were less aware of physical discomfort and disease aggravation. In addition, ethnic minority residents were worse off than those of Han nationality. In terms of health indicators, despite ethnic minorities having a higher probability of chronic disease occurrence than the Han residents (35.1% vs. 9.1%, *p* = 0), they had the same self-reported health assessment probability (*p* = 0.598) and the same probability of having an impairment or disability that restricted their daily activities (*p* = 0.094). Thus, ethnic minority residents were shown to be significantly less aware of disease prevention and treatment than those of Han nationality. Table 2 shows that the probability of visiting the doctor due to heat-related ailments was significantly lower for minority residents than for Han residents (17.5% vs. 35%, *p* = 0). Furthermore, there were no significant inter-group differences regarding taking adaptive actions or asking for help.

**Figure 2:**
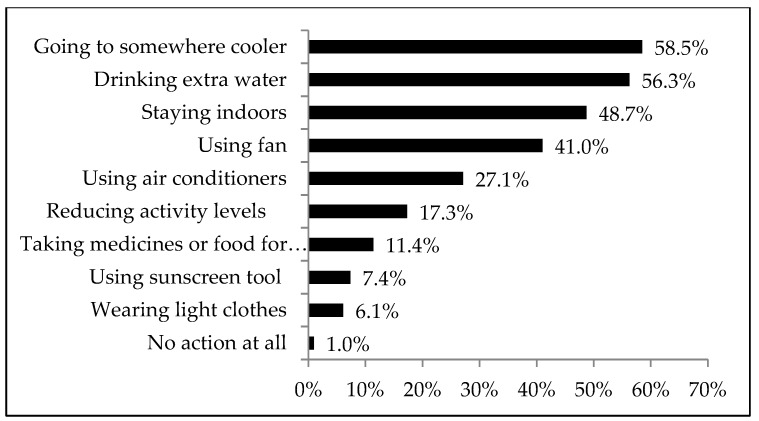
Choices of the participants’ coping behaviors during hot weather.

In terms of coping behaviors, 58.5% of respondents stated that in hot weather, they choose to move somewhere cooler, 56.3% drink extra water, 48.7% stay indoors, 41% use a fan, 27.1% use an air conditioner, 7.4% use sunscreen, and 6.1% wear light-colored clothes (Figure 2).

### 3.2. Logistic Regression Results

The logistic models indicated that ethnic minority, residence time, outdoor working hours, and health status could significantly influence the perceptions of the participants. Those who were of Miao or Tujia ethnic minority or had a shorter residence time were more likely to be aware of the warm temperature and the associated health risks. Those who worked or stayed outdoors for less than seven hours a day in July and August were more likely to have perceptions of associated health risks. Those in the Han group were more likely to have perceptions of physical discomfort and aggravated disease during hot weather. Those who had poorer health status were more likely to have perceptions of aggravated disease during hot weather (Table 3).

Table 4 shows that these perceptions could significantly affect responses. Those who were aware of warm temperature were more likely to see a doctor if they perceived physical discomfort (OR = 2.596), take adaptive actions (OR = 6.021), and ask for help during hot weather (OR = 2.651). Those who perceived the aggravation of a disease were more likely to see a doctor (OR = 20.347) but were less likely to ask for help during hot conditions (OR = 0.314). Those who perceived physical discomfort during the heat were more likely to take adaptive actions (OR = 5.974) and ask for help (OR = 3.677).

## 4. Discussion

This paper aimed to study the perceptions and responses related to health risks from hot weather in ethnic minority area in southwest China, by analyzing representative samples from Pengshui Miao and the Tujia Autonomous County in Chongqing. According to the meteorological data, Pengshui County belongs to the mid-tropical warm monsoon climate zone, with an annual average temperature of 17.5 °C. In recent years, the number of hot weather days and the length of heatwave in July and August have increased year by year. In 2018, 44 days had a temperature above 35 °C, and the highest temperature was 42 °C. This is the hottest year recorded, except for 2011 and 2013. An analysis of 624 respondents obtained in a multi-stage sample of the county found that 80% of residents felt increasing temperatures in recent years and 79% thought hot weather would be a health threat. Almost all survey participants reported a response to hot weather: 58.5% choose to go to somewhere cooler, 56.3% drink extra water, 48.7% stay indoors, and 41% use a fan. This indicates that residents in the survey area have a high awareness of hot weather and its related health risks, which is consistent with the findings of some other scholars [21,22]. The results of this study showed that ethnic minority residents and Han residents have significant differences in their health-related perceptions of hot weather, with ethnic minority participants (61.2% of the study population) having a higher awareness of warm temperature (*p* <0.001) and the associated health risks (*p* <0.001). This conclusion is consistent with the research of other scholars. According to a study in Australia, ethnic minorities and immigrants are more susceptible to hot weather [23]. A previous study on racial differences in calorie-related mortality in four U.S. cities showed that race and immigration status are also important factors in heat perception [24]. According to the survey, the perception of climate change by ethnic minority residents was based on intuitive cognition and judgments gradually formed by traditional knowledge, which is reflected in their life production habits and cultural traditions. The influence of traditional knowledge on the perception of minority residents has also been mentioned in other scholars’ research. Zander (2013) assessed the views of Aboriginal people in coastal communities in northern Australia on the impacts of climate change and their possible precautionary responses to both sea level rise and a potential increase in the intensity of tropical cyclones in coastal communities [25]. All respondents had heard about climate change. At the same time, ethnic minority participants had a lower perception of physical discomfort (*p* <0.001) and aggravated diseases (*p* = 0.001). When the hot weather eroded their own health, the probability of visiting a doctor by minority residents was significantly lower than that of Han residents (*p* = 0), which may be because they are more likely to “forget” or endure the effects of hot weather. In addition, minority residents had similar high-temperature responses to those of the Han people. Health status was also shown to significantly affect the local residents’ perceptions related to health risks from hot weather. Those who had poorer health status were more likely to have perceptions of aggravated disease during hot weather. Those with worse health status included individuals with a self-reported poor health status (12% of participants), impairment or disability with restricted movement (10.3%), or chronic disease (25%), which is basically consistent with the research of other scholars [21,22]. The low prevalence of self-reported poor health compared to chronic disease indicates that the respondents neglect chronic diseases and need to improve related prevention and control strategies.

Factors such as local residence time and outdoor working hours had an impact on the local residents’ perceptions related to health risks from hot weather. Those who had a shorter residence time were more likely to be aware of warm temperature and associated health risks. Therefore, the shorter the local residence time is, the greater the occupational mobility of the respondents is, the lower the tolerance for local climate change is, and the stronger the perception of warm temperature. Those who worked or stayed outdoors for less than seven hours a day in July and August were more likely to have perceptions of associated health risks. This may be because outdoor workers are more susceptible to the effects of hot weather [26].

In the study of the relationship between high-temperature health risk perceptions and responses, this paper found that perception of warm temperature affected almost all responses including seeing a doctor if physical discomfort is perceived during hot weather, taking adaptive actions, and asking for help. The stronger the perception of warm temperature, the more positive the response behavior, and this conclusion is consistent with the research of other scholars [22,27]. In addition, those who perceived aggravated diseases were more likely to see a doctor (OR = 20.347) but were less likely to ask for help during hot weather (OR = 0.314). Those who perceived physical discomfort during hot weather were more likely to take adaptive actions (OR = 5.974) and ask for help (OR = 3.677).

## 5. Conclusions

Our study analyzed rural residents’ perception and response to health risks from hot weather in ethnic minority areas in southwest China. Despite the relatively high level of risk perception, the study population had a very low level of willingness to see a doctor, especially the ethnic minority residents. We also found that ethnic minority, residence time, outdoor working hours, and health status affected perceptions, subsequently influencing coping behaviors. Our findings have significant implications for the development of policies and health education and promotion programs for ethnic minorities in southwest China to promote good health during future hot weather events.

## Figures and Tables

**Table 1:** Main characteristics of the participants. CI: confidence interval.

Variables	Proportion/Mean	95% CI
Ethnic minority	61.2%	(57.4%, 65.1%)
Age	43	(41, 44)
Female	33.7%	(29.9%, 37.4%)
Residence time	39	(37, 40)
Education beyond high school	58.2%	(54.3%, 62.1%)
Married	73.6%	(70.1%, 77%)
Employment	34.3%	(30.6%, 38%)
Working or staying outdoors for no fewer than 7 hours a day in July and August	36.2%	(32.4%, 40%)
Health status		
Self-reported a poor health status	12%	(9.4%, 14.6%)
Impairment or disability with restricted movement	10.3%	(7.9%, 12.6%)
Chronic disease	25%	(21.6%, 28.4%)

**Table 2:** Perceptions and responses among the different ethnic minority groups.

Variables	Ethnic Group	Proportion	*p*-Value
Perception of warm temperature	Total	80%	
	Ethnic minority	84.6%	
	Han minority	73%	<0.001
Perception of associated health risks	Total	79%	
	Ethnic minority	85%	
	Han minority	69.4%	<0.001
Perception of physical discomfort during hot weather	Total	92%	
	Ethnic minority	88.7%	
	Han minority	97.6%	<0.001
Perception of aggravated disease during hot weather	Total	35%	
	Ethnic minority	31.4%	
	Han minority	40.5%	0.020
Seeing a doctor following physical discomfort during hot weather	Total	24.4%	
	Ethnic minority	17.5%	
	Han minority	35%	<0.001
Taking adaptive actions during hot weather	Total	98.7%	
	Ethnic minority	98.4%	
	Han minority	99.2%	0.422
Asking for help	Total	91%	
	Ethnic minority	90.6%	
	Han minority	91.7%	0.622

**Table 3:** Logistic regression results on perceptions.

Models	Odds Ratio	Robust Std. Err.	*p*-Value	95%CI
**Model 1—Perception of warm temperature**				
Ethnic minority	2.395	0.523	0	(1.561, 3.673)
Residence time	0.987	0.006	0.024	(0.976, 0.998)
**Model 2—Perception of associated health risks**				
Ethnic minority	3	0.662	0	(1.948, 4.624)
Residence time	0.983	0.006	0.005	(0.972, 0.995)
Working or staying outdoors for no less than 7 hours a day in July and August	0.431	0.089	0	(0.287, 0.647)
**Model 3—Perception of physical discomfort during hot weather**				
Ethnic minority	0.2	0.089	0	(0.084, 0.478)
**Model 4—Perception of aggravated disease during hot weather**				
Ethnic minority	0.514	0.099	0.001	(0.352, 0.751)
Self-reported poor health status	2.073	0.571	0.008	(1.208, 3.555)
Having impairment or disability with restricted movement	2.490	0.714	0.001	(1.419, 4.368)
Having a chronic disease	2.502	0.552	0	(1.623, 3.857)

**Table 4:** Logistic regression results for the responses.

Models	Odds Ratio	Robust Std. Err.	*p*-Value	95%CI
**Model 1—Seeing a doctor if physical discomfort is perceived during hot weather**				
Perception of warm temperature	2.596	0.919	0.007	(1.297, 5.197)
Perception of aggravated disease during hot weather	20.347	5.075	0	(12.479, 33.174)
**Model** **2—Taking adaptive actions during hot weather**				
Perception of warm temperature	6.021	4.5	0.016	(1.392, 26.052)
Physical discomfort during heat	5.974	4.56	0.019	(1.339, 26.65)
**Model** **3—Asking for help**				
Perception of warm temperature	2.651	0.841	0.002	(1.423, 4.94)
Physical discomfort during heat	3.677	1.607	0.003	(1.561, 8.66)
Perception of aggravated disease during hot weather	0.314	0.1	0	(0.169, 5.755)
